# SomatoSim: precision simulation of somatic single nucleotide variants

**DOI:** 10.1186/s12859-021-04024-8

**Published:** 2021-03-06

**Authors:** Marwan A. Hawari, Celine S. Hong, Leslie G. Biesecker

**Affiliations:** Medical Genomics and Metabolic Genetics Branch, National Human Genome Research Institute, National Institutes of Health, Bethesda, MD USA

**Keywords:** Single nucleotide variants, SNV, Simulation, Somatic variants, Mosaicism

## Abstract

**Background:**

Somatic single nucleotide variants have gained increased attention because of their role in cancer development and the widespread use of high-throughput sequencing techniques. The necessity to accurately identify these variants in sequencing data has led to a proliferation of somatic variant calling tools. Additionally, the use of simulated data to assess the performance of these tools has become common practice, as there is no gold standard dataset for benchmarking performance. However, many existing somatic variant simulation tools are limited because they rely on generating entirely synthetic reads derived from a reference genome or because they do not allow for the precise customizability that would enable a more focused understanding of single nucleotide variant calling performance.

**Results:**

SomatoSim is a tool that lets users simulate somatic single nucleotide variants in sequence alignment map (SAM/BAM) files with full control of the specific variant positions, number of variants, variant allele fractions, depth of coverage, read quality, and base quality, among other parameters. SomatoSim accomplishes this through a three-stage process: variant selection, where candidate positions are selected for simulation, variant simulation, where reads are selected and mutated, and variant evaluation, where SomatoSim summarizes the simulation results.

**Conclusions:**

SomatoSim is a user-friendly tool that offers a high level of customizability for simulating somatic single nucleotide variants. SomatoSim is available at https://github.com/BieseckerLab/SomatoSim.

## Background

Somatic mutations can occur in the DNA of a single cell in an individual, and with subsequent clonal expansion, lead to somatic mosaicism. Moreover, somatic single nucleotide variants (SNVs) are the most common form of somatic variants. Due to their association with cancer and rare diseases [1, 2], growing interest in the detection of these variants has led to the development of case–control matched and unmatched somatic variant calling tools [for review, see [3]]. However, the lack of truth datasets to accurately assess variant calling has increased the usage of somatic variant simulation to validate and benchmark these methods.

Currently, there are two general approaches to simulating somatic variants in next-generation sequencing (NGS) data. In the first approach, somatic variants are simulated by generating entirely synthetic reads based on a reference genome. The main limitation of this approach is that error profiles resulting from real sequencing experiments cannot be accurately represented and can only be estimated. In the second approach, experimentally generated reads from BAM files are used to create simulated somatic variants. Depending on the specific simulation tool, this second approach can be implemented in a variety of ways including creating an admixture of multiple BAM files with known heterozygous sites or directly introducing simulated variants into the reads of a BAM file [4, 5]. Directly introducing variants into experimentally generated reads not only preserves error profiles, but also facilitates the study of a large number of variants across a range of VAFs and sequencing depths [5]. However, there is still a need for a tool that enables precise simulation of somatic SNVs with a greater degree of customizability.

To address this need, we developed SomatoSim, a simple to use tool that provides nucleotide-level specification for simulated SNVs in experimentally generated reads and provides detailed output reports to document the results. Requiring only a BAM file and a BED file, SomatoSim provides the user precise control over where to simulate SNVs, which VAF values to simulate, and the desired depths of coverage. Further, SomatoSim is the only tool that allows the users to set specific parameters such as a read mapping quality (MQ) threshold, a position base quality (BQ) threshold, and a minimum distance between randomly simulated SNVs, enabling the generation of highly specific datasets that suit individualized user needs. The ability to define a MQ or BQ threshold allows the users to avoid simulating variants in positions with low MQ and BQ scores, as such scores may indicate systematic errors and bias occurring in sequencing or mapping [6]. Additionally, SomatoSim introduces simulated SNVs directly into the BAM file reads, preserving the real sequencing environment and experiment specific error. Moreover, SomatoSim models the original strand distribution of a position selected for simulation and mutates reads to reflect the strand ratio of that position. This is important as local mapping can lead to strand bias in a given position [7]. Simulating SNVs with uniform or completely random strand distributions may not accurately reflect the true mapping bias at a given position. Finally, SomatoSim minimizes pre and post-simulation workload for the user, as it requires little set-up, is highly flexible with the input data, and provides extensive reports on the simulation results. Through these features, SomatoSim addresses the need for a user-friendly somatic variant simulation tool that enables precise, nucleotide-level, simulated SNV customization in experimentally generated reads.

## Implementation

**Fig. 1:**
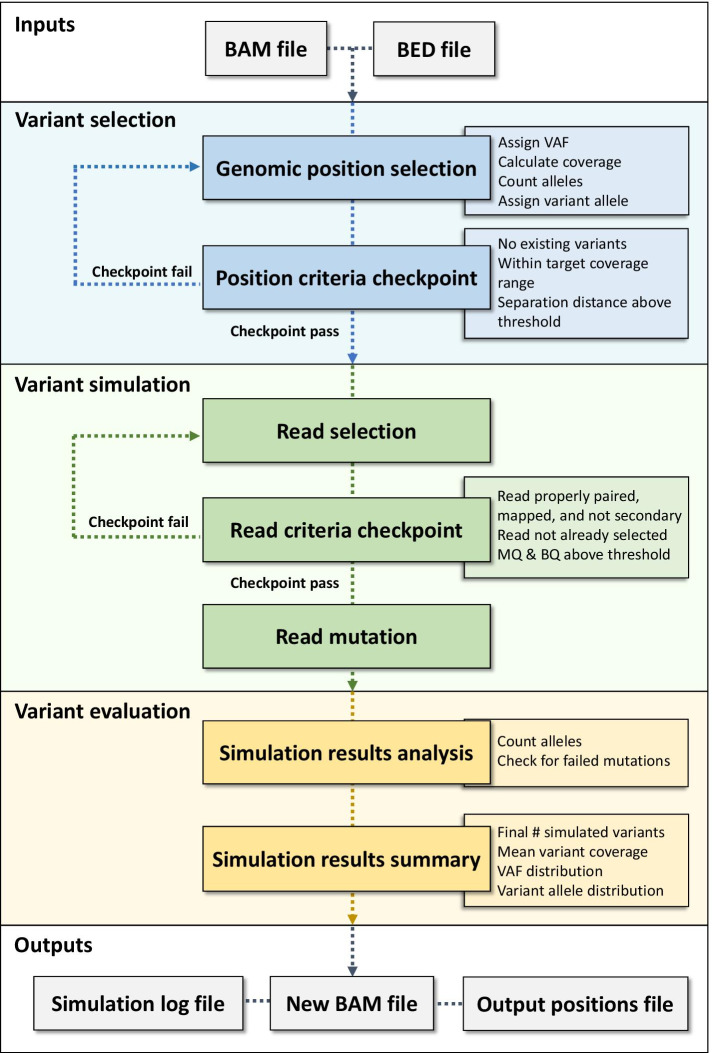
Schematic overview of the SomatoSim workflow

SomatoSim works in three main stages: a variant selection stage, a variant simulation stage, and a variant evaluation stage (Fig. 1). In the variant selection stage, genomic positions where variants will be introduced are selected from the input BED file. In the variant simulation stage, reads associated with the selected positions are mutated and a new BAM file is generated. Finally, in the variant evaluation stage, the results of the variant simulation stage are analyzed and reported to the user.

### Variant selection

There are two distinct variant selection methods: random variant selection and user-specified variant selection. For random variant selection, a single genomic position is randomly chosen from a genomic range in the input BED file. Following this selection, a random variant allele fraction (VAF), whose upper and lower bounds are defined by the user, is assigned to that position. The depth of coverage and raw allele counts are then computed for that position using SAMtools [8] mpileup with the input MQ and BQ settings. From the allele counts, an observed genotype is determined, and the position is randomly assigned a variant allele. Finally, the integer number of reads that must be mutated to achieve the desired VAF is calculated as the product of the assigned VAF and coverage. The limitation of this approach is that the lowest possible simulated VAF and the resolution of possible VAFs are dependent on the depth of coverage in the user’s input BAM file.

For user-specified variant selection, the user can specify single genomic positions, a desired VAF for every position, and a desired variant allele for every position. SomatoSim will automatically detect this information in the BED file and enter the user-specified variant selection mode. The process for user-specified variant selection is largely the same as the process for random variant selection; however, the user-specified VAF value and user-specified variant allele are assigned to its corresponding position in the BED file.

For both methods, potential positions are checked for several criteria to determine if they are suitable for variant simulation. First, positions that already have multiple alleles (above the user input BQ and MQ) are not considered for variant simulation. Second, if a target coverage and coverage tolerance are specified by the user, only positions within the target coverage range are retained. Third, the number of bases between potential positions must be greater than or equal to the minimum separation value specified by the user. In user-specified variant selection, a specified variant allele must be different than the observed genotype. Additionally, SomatoSim checks if the calculated number of reads to mutate for a position is greater than zero. This is calculated as the product of the coverage and associated VAF, rounded to the nearest whole number. Positions that require zero reads to be mutated after rounding are not selected. Positions that pass the user input criteria are added to a list of genomic positions where variants will be introduced, while positions that do not pass the criteria are not considered for simulation.

### Variant simulation

In the variant simulation stage, SomatoSim builds a bank of reads to mutate, writes variants to those reads, and generates a new BAM file with the simulated variants. There are two main processes in the variant simulation stage: read selection and read mutation.

In the read selection process, reads that will be mutated are selected for every genomic position chosen in the variant selection stage. Several criteria are assessed to ensure that the read is suitable for mutation (Fig. 1). First, the read must be properly paired and mapped and must not be a secondary read. Second, the read MQ and the allele BQ in the read must be above the input threshold values. Last, a read can be selected only once and cannot be re-selected for variant simulation in another position. This is to minimize the generation of low-fidelity reads, as multiple real variants occurring within a single short sequencing read (< 150 bp) are expected to be uncommon [9, 10] (Additional file 1: Table S1). Also during the read selection process, SomatoSim models the strand distribution of the real data at a given position so that the strand distribution of variant reads reflects the strand distribution of the original reads at that same position.

During the read mutation process, SomatoSim creates a new BAM file containing the simulated variants. In this step, a read selected for mutation is replaced with the same read but containing the variant allele. The reads are not realigned, as we observed that > 99.5% of the mutated reads correctly re-mapped to the same location (Additional file 1: Table S1). With this approach, unexpected changes in coverage and VAF are minimized, the total run time is reduced, and the read and the variant allele retain their original read MQ and BQ, respectively. However, the < 0.5% of mutated reads that do not correctly re-map present a limitation of this method and may be an important consideration for researchers, especially when examining regions with high sequence similarity content.

### Variant evaluation

The variant evaluation stage of SomatoSim analyzes and summarizes the results of the variant simulation stage by providing the user with a suite of metrics to aid the interpretation and validation of those simulation results (Fig. 1). In this stage, SomatoSim will count alleles in the new BAM file and check for any failed mutations. A failed mutation will occur if there are not enough criteria-passing reads at a given position to achieve a VAF that is within the input range of VAF values. If the resulting VAF at a failed mutation position is not zero, then that position will still have some non-zero number of mutant reads in the new BAM file. Shortages in criteria-passing reads may occur if the target area is already saturated with other simulated variants, if the BAM file has a low depth of coverage, or if the MQ of the reads and the BQ of the bases are lower than the thresholds.

SomatoSim produces the following output files: a new BAM file containing the simulated variants, the associated index file, a log file with detailed metrics from each stage of SomatoSim, and a BED formatted text file that reports relevant details for each simulated variant. These files allow the user to evaluate the results of the simulation and verify that the desired mutations were truly introduced. The metrics in the log file include the number of positions selected for simulation, the average coverage of these positions, the required number of reads to mutate, the actual number of reads mutated, the final number of successfully simulated variants, the number of failed mutations, and the VAF and variant allele distributions for the final simulated variants. The metrics in the BED formatted text file include every position where simulated SNVs were introduced and the corresponding VAF, coverage, and variant allele.

### Test data

The test BAM file is derived from the National Institute of Standards and Technology (NIST) Genome in a Bottle (GIAB) NA12878 HiSeq 300X BAM file [11]. The exon regions used in the test BED file were derived from the GENCODE Release 27 (GRCh37) comprehensive gene annotation gff3 file [12].

To create the test BED file, 12 exonic regions for each chromosome (including both X and Y) were randomly selected from the GENCODE exon annotation, resulting in a total of 159,709 positions. We used BEDtools pairToBed [13] to intersect the selected exonic regions with the GIAB BAM file and created a sub-sampled BAM file known to contain the exonic regions in the test BED file. The BAM file was then re-aligned to the hs37d5 reference genome using SAMtools fastq and BWA-mem and had duplicate reads marked by Picard MarkDuplicates (http://broadinstitute.github.io/picard).

The test_BED_user.bed file contains 190 genomics positions and was created by randomly selecting a single genomic position from each genomic range in the BED file and assigning it a VAF and variant allele.

## Results and discussion

### General performance

To understand the general performance of SomatoSim, we simulated varying numbers of SNVs, from 10 to 10,000, in the provided test BAM file using the test BED file. With SomatoSim, the user can choose to down-sample the input BAM file to a desired average coverage value. This can help the user better target a specific depth of coverage for the simulated variants. For this case, we used random variant selection and set the VAF range from 0.01 to 0.10, down-sampled the BAM file to 100X, and targeted positions with 100X coverage. To evaluate the results of the simulation, we recorded the run time and the mutation yield. The mutation yield was defined as the ratio of the number of successfully simulated SNVs (N_success_) to the total number of SNVs defined for simulation (N_simulate_).

For these simulations, the provided test BED file contained a total of 159,709 genomic positions. The average coverage of the down-sampled BAM file over the BED file positions was 100 (when using the default BQ threshold of 20), and the down-sampled BAM file contained a total of 118,795 reads.

**Fig. 2:**
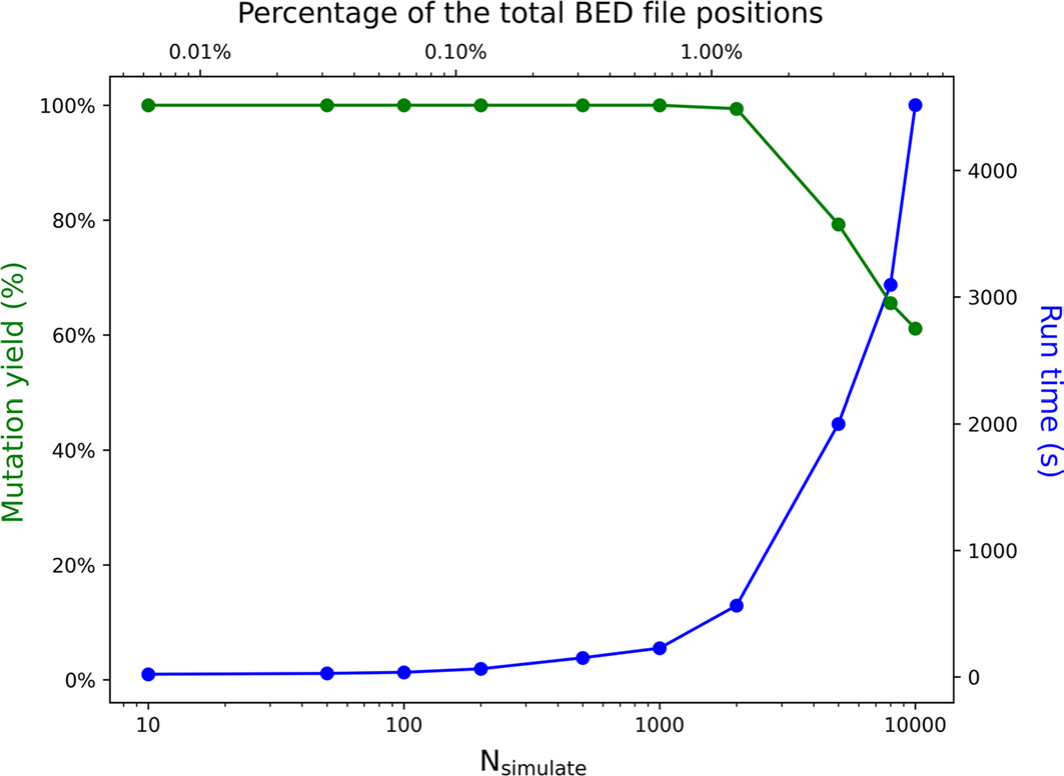
Evaluation of mutation yield and run time for SomatoSim. N_simulate_ is the total number of SNVs defined for simulation

Under these conditions, we observed a 100% mutation yield for values of N_simulate_ between 10 and 1000 (0.006–0.626% of the total BED file positions). The mutation yield first decreased below 100% when N_simulate_ > 1000 and fell below 80% when N_simulate_ = 5000 (3.131% of the total BED file positions) (Fig. 2 and Additional file 1: Table S2). Finally, when N_simulate_ = 10,000 (6.261% of the total BED file positions), the mutation yield approached 60%. This decrease in mutation yield was expected and demonstrates a limitation of SomatoSim that results from only allowing a criteria-passing read to be selected once for mutation. More specifically, as the number of simulated SNVs increases, the availability of criteria-passing reads decreases, thus limiting the total possible locations for simulating SNVs and increasing the number of failed mutations. As a result, the mutation yield is dependent on the percentage of positions in the BED file selected for mutation, the distribution of genomic intervals in the BED file, the BAM file’s depth of coverage, and the read length. Therefore, SomatoSim is not recommended for simulating SNVs in BAM files that were generated by long-read sequencing platforms.

To evaluate the speed of SomatoSim, we also examined the run time when using 1 CPU and 1 GB of memory (Fig. 2). Expectedly, the run time increased as N_simulate_ increased. This is due to increased time searching for criteria-passing positions during the variant selection stage and criteria-passing reads during the variant simulation stage. Other factors that may affect the run time include the quality of the BAM file, the size of the BAM file, the sequencing depth, and the distribution of the BED file positions across the BAM file. Under these considerations, SomatoSim was designed for use with exome or custom capture BAM files.

### Comparison to existing variant simulation tools

As previously described, somatic variants are currently being simulated using two general approaches. In the first approach, variants are introduced into a reference genome and synthetic reads containing the variants are generated using a variety of error modeling techniques. The main limitation of this approach is that it fails to capture the instrument- and experiment-specific sequencing error of actual NGS data. Within this category are tools such as VarSim [14], SInC [15], DWGSIM (https://github.com/nh13/DWGSIM), and GemSim [16]. Furthermore, while these tools can all simulate somatic SNVs, they do not provide the user with the degree of customization that we think is important (Table 1). For example, VarSim currently restricts possible somatic variants to only those found in the COSMIC database [2], SInC only allows users to control the percentage of the reference genome that will contain simulated SNVs, and DWGSIM limits users to a single VAF for all simulated somatic SNVs. In this category, GemSim is most similar to SomatoSim insofar as GemSim allows users to specify the variant allele and VAF for each position in an extra input file.

**Table 1:** A comparison of features between SomatoSim and existing somatic variant simulation tools

	SomatoSim	VarSim	SInC	DWGSIM	GemSim	tHapMix	Xome-Blender	BAMSurgeon
Variants introduced into experimentally generated reads	✓	X	X	X	X	✓	✓	✓
User definable VAFs	✓	X	X	X	✓	X	X	✓
User definable depth of coverage	✓	✓	✓	✓	X	✓	✓	✓
MQ and BQ control	✓	X	X	X	X	X	X	X
User definable separation distance between randomly simulated SNVs	✓	X	X	X	X	X	X	X
Strand distribution modeling	✓	X	X	X	X	X	X	X
Types of somatic variants	SNV	SNV, Indel, SV	SNV, Indel, CNV	SNV, Indel	SNV	SNV, CNV	SNV, Indel, CNV	SNV, Indel, CNV, SV
Output data type	BAM	FASTQ	FASTQ	FASTQ	FASTQ	BAM	BAM	BAM

In the second approach to simulating somatic variants, variants are introduced into experimentally generated reads, eliminating the need for artificial error modeling. Within this category are tools such as tHapMix [17], Xome-Blender [4], and BAMSurgeon [5]. Although tHapMix has a great number of features to simulate tumor characteristics, including clonal evolution simulation, it limits the potential SNV simulation sites to those already identified in an input VCF file and does not allow users to specify an exact VAF for each simulated SNV.

Xome-Blender, which simulates somatic variants through a method of BAM file sub-sampling and mixing, allows users to specify the number of SNVs to simulate, but not the precise positions. In this category, BAMSurgeon is most similar to SomatoSim, as it generates simulated variants by directly modifying reads in an input BAM file and allows users to input another file to specify the variant allele and VAF for each position. The customizability features of SomatoSim extend further and allow the user to define not only the precise position, variant allele, VAF, and coverage for SNVs, but also the distance between randomly simulated SNVs and the MQ and BQ threshold values for SNVs. Additionally, SomatoSim has a built-in down-sampling feature to simplify any BAM file pre-processing and streamline the generation of many simulated datasets with different depths of coverage. SomatoSim also automatically models the (forward and reverse) strand distribution of a given position to ensure that the strand distribution of the reads containing the simulated variant reflects the original strand distribution. This is especially important because a uniform or random strand distribution may not accurately capture the true sequencing environment and may also affect downstream variant calling tools that account for the strand distribution when making a call. Finally, unlike BAMSurgeon, SomatoSim does not re-align reads and is thus able to minimize unexpected changes in coverage and VAF while greatly improving run time. When using the test_BED_user.bed file, the test BAM file, the default input options for SomatoSim, and the similar corresponding options for BAMSurgeon, we found that SomatoSim finished in approximately 44 s while BAMSurgeon finished in approximately 56 min. This improvement in run time facilitates the generation of simulated datasets without substantial time and resource investment.

### Case study

To demonstrate usage of SomatoSim, we used the provided test data BAM and BED files to simulate 1,000 SNVs from 0.01 to 0.10 VAF at a 100X target coverage. During the read selection process, a total of 5,981 positions were checked as potential simulation positions before all 1,000 desired positions that passed the user input criteria were selected. The “--verbose” option details positions that did not pass the user input criteria and were not selected. For this example, 282 positions already had an existing variant, 4,255 positions did not meet the position coverage criteria of 100X coverage with the selected 10% coverage tolerance option (“--coverage-tolerance”), and 441 positions were not separated from a previously selected position by at least one position (Table 2). Since SomatoSim checks if the calculated number of reads to mutate for a position is greater than zero, three positions that would have required zero reads to be mutated (after rounding) were also not selected. Users can use this report to determine the stringency of the input parameters. For this case study simulation, the number of positions that do not pass the criteria could potentially be decreased by increasing the coverage tolerance. For those 1000 selected positions, a total of 5,294 reads were selected and mutated, resulting in variants with VAF values between 0.01 and 0.10 in our 100X down-sampled output BAM file. The total run time using 1 GB of memory and 1 CPU was approximately 3.7 min (Additional file 1: Table S2).

**Table 2:** Number of positions that failed the criteria for position selection

Failed criterion	Number of positions
No existing variant	282
Within target coverage	4255
Minimum SNV separation distance	441
Number of reads to mutate is greater than zero	3

We then used this simulated dataset to illustrate how SomatoSim can be used for evaluating the performance of variant calling tools. In this case, we examined the sensitivity of LoFreq [18], a widely used somatic variant caller, across a range of coverages and VAFs using several BAM files generated by SomatoSim. We used SAMtools to merge the 100X BAM file described above with itself and generate 200X, 400X, 600X, and 800X BAM files, all containing 1,000 simulated SNVs with VAFs between 0.01 and 0.10. Finally, we ran LoFreq with the recommended default settings and computed sensitivity as the ratio of the number of simulated SNV calls to the total number of simulated SNVs.

**Fig. 3:**
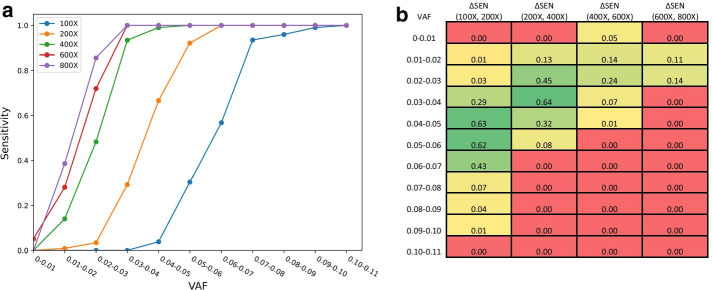
Evaluation of sensitivity in LoFreq using simulated SNVs generated by SomatoSim. **a** Sensitivity curve for different depths of coverage and VAFs tested. **b** Heatmap of the change in sensitivity (ΔSEN) across depth of coverage. Green indicates substantial improvement in sensitivity and red indicates no improvement

As expected, we observed a general increase in sensitivity as the coverage and VAF increased. However, we found that for simulated SNVs with very low-level VAFs (VAF < 0.01), the sensitivity generally did not increase with an increase in coverage, suggesting the limit of detection for LoFreq (Fig. 3a and Additional file 1: Table S3). Understanding the limit of detection will enable researchers to choose the most appropriate variant calling tool for their NGS projects.

When we examined the change in sensitivity (ΔSEN) due to increasing the depth of coverage, we found that for simulated SNVs with VAF ≥ 0.05, increasing the coverage from 100 to 200X resulted in a greater improvement in sensitivity than when increasing the coverage from 200 to 400X (Fig. 3b). Additionally, for simulated SNVs with VAF < 0.03, increasing the coverage from 200 to 400X resulted in a greater improvement in sensitivity than when increasing the coverage from 100 to 200X.

More broadly, we observed the greatest improvements in sensitivity from 100 to 400X (Fig. 3b). The sensitivity for simulated SNVs with 0.03 ≤ VAF < 0.04 increased by 0.93 when the coverage increased from 100 to 400X. We also found that increasing the coverage from 400 to 800X only improved the sensitivity by 0.07. Furthermore, all simulated SNVs between 600 and 800X coverage with a VAF ≥ 0.03 were called by LoFreq. This suggests that sequencing above 600X coverage is not necessary for calling SNVs with VAF ≥ 0.03 when using LoFreq. Similarly, for all simulated SNVs with VAF ≥ 0.04, the sensitivity increased only by 0.01 when the coverage increased from 400 to 800X (Fig. 3b). This indicates that increasing coverage above 400X is most beneficial for calling SNVs with 0.01 ≤ VAF < 0.03 when using LoFreq.

In this case study, we used LoFreq to call simulated SNVs from a dataset generated by SomatoSim and analyzed how sequencing depth and VAF affect the variant calling performance. In doing so, we have also demonstrated how simulated datasets generated by SomatoSim can be used to examine the strengths and weaknesses of a variant calling tool and provide a means for benchmarking the performance of variant calling tools, algorithms, and pipelines.

## Conclusion

In this paper, we report SomatoSim, a tool for simulating somatic SNVs in experimentally generated NGS data. With SomatoSim, we have addressed the need for a user-friendly tool that can provide users with the customizability and flexibility to fine-tune various somatic SNV properties, allowing for the precise simulation of SNVs for downstream analyses.

Using SomatoSim to generate a dataset with simulated SNVs enables researchers to evaluate the strengths and weaknesses of variant calling tools and benchmark variant calling performance. By doing so, researchers developing novel somatic variant calling methods can understand how their new method compares with existing methods. Additionally, researchers implementing somatic variant calling pipelines can determine which tools would be most appropriate for their studies. Finally, by understanding how factors like VAF and sequencing depth affect variant calling, researchers can better tailor their sequencing experiments for their somatic variant calling pipelines.

Through an example simulation and detailed case study, we demonstrated how SomatoSim can be used to generate simulated SNVs in experimentally generated NGS data to evaluate the performance of a variant calling tool. The flexibility, precision, and ease of use provided by SomatoSim enables the efficient generation of simulated datasets for studying somatic SNVs and improving somatic SNV calling methods.

## Availability and requirements

• Project name SomatoSim.

• Project home page https://github.com/BieseckerLab/SomatoSim

• Operating system Platform independent.

• Programming language Python 3.

• Other requirements Python v3.6.8, NumPy v1.16.2, Pandas v0.25.1, Matplotlib v3.1.1, Pysam v0.15.0, SAMtools v1.9

• License This software is a United States Government Work. Anyone may use the software on a worldwide and royalty-free basis for any purpose and anyone may reproduce and prepare derivative works without restriction. Although all reasonable efforts have been taken to ensure the accuracy and reliability of the software, the National Human Genome Research Institute (NHGRI), National Institutes of Health (NIH) and the U.S. Government do not and cannot warrant the performance or any results that may be obtained by using this software. NHGRI, NIH and the U.S. Government disclaim all warranties as to performance, merchantability or fitness for any particular purpose. No indemnification is intended or provided by the US government.

This is software was developed by Marwan A. Hawari, Celine S. Hong, and Leslie G. Biesecker at the National Human Genome Research Institute (NHGRI), National Institutes of Health (NIH). Please include proper attribution of NHGRI as the developer of this program and include a link to the following [https://github.com/BieseckerLab/SomatoSim] in all publications and other public disclosures that reference the program and/or include data or research results that were generated using the program.

• Any restrictions to use by non-academics: None.

## Supplementary Information

**Additional file 1: Table S1**. Evaluation of variant read re-alignment. **Table S2**. General performance metrics of SomatoSim. **Table S3**. Sensitivity values from the case study.

## Data Availability

The datasets generated and analyzed during this study are available on the SomatoSim GitHub repository (https://github.com/BieseckerLab/SomatoSim).

## Abbreviations

BED: Browser extensible data
SNV: Single nucleotide variant
NGS: Next-generation sequencing
MQ: Mapping quality
BQ: Base quality
VAF: Variant allele fraction
NIST: National Institute of Standards and Technology
GIAB: Genome in a Bottle
SV: Structural variant
CNV: Copy number variant
ΔSEN: Change in sensitivity
