# Supplementary material for: SomatoSim: precision simulation of somatic single nucleotide variants
Source: BMC Bioinformatics. 2021 Mar 6;22:109. doi: 10.1186/s12859-021-04024-8 (PMC7936459; doi:10.1186/s12859-021-04024-8)
Supplement: Supplementary file 1 — Additional file 1: Table S1. Evaluation of variant read re-alignment. Table S2. General performance metrics of SomatoSim. Table S3. Sensitivity values from the case study. [file 12859_2021_4024_MOESM1_ESM.docx]

**Supplementary information**

**SomatoSim re-alignment tests**

The recommended input for SomatoSim is a processed, analysis ready BAM file. This allows users to directly simulate SNVs in their own sequencing files, preserving the experimental and bioinformatics processing environment. With this approach, SomatoSim does not re-align reads after introducing SNVs. By only allowing one SNV per read and only introducing SNVs in primary reads, it is expected that alternate alignment would be minimal. To test this, we simulated varying numbers of SNVs with the default parameters using the test data BAM and BED files. Then, we deconstructed the resulting BAM files into FASTQ files using SAMtools [1] fastq, re-aligned them using BWA-MEM [2], and compared the number of variant reads at our simulated positions before and after re-alignment using the same default MQ and BQ threshold values. The results in Supplementary Table S1 show that the percentage of successfully re-aligned variant reads is greater than 99.5% regardless of the number of simulated variants.

**Supplementary Table S1 Evaluation of variant read re-alignment**

| Number of simulated SNVs | Number of mutated reads before re-alignment | Number of successfully re-aligned mutant reads | Percentage of successfully re-aligned mutant reads |
| --- | --- | --- | --- |
| 500 | 14425 | 14372 | 99.63 |
| 1000 | 28884 | 28768 | 99.60 |
| 1500 | 43330 | 43141 | 99.56 |
| 2000 | 57999 | 57753 | 99.58 |

**Supplementary Table S2 General performance metrics of SomatoSim**

| N_simulate_ | Percentage of BED file positions attempted to mutate | N_success_ | Mutation yield (%) | Run time (s) |
| --- | --- | --- | --- | --- |
| 10 | 0.00626 | 10 | 100 | 20.76 |
| 50 | 0.03131 | 50 | 100 | 27.11 |
| 100 | 0.06261 | 100 | 100 | 36.18 |
| 200 | 0.12523 | 200 | 100 | 63.50 |
| 500 | 0.31307 | 500 | 100 | 150.85 |
| 1000 | 0.62614 | 1000 | 100 | 227.01 |
| 2000 | 1.25228 | 1988 | 99.40 | 564.26 |
| 5000 | 3.13069 | 3963 | 79.30 | 1999.11 |
| 8000 | 5.00911 | 5243 | 65.54 | 3095.93 |
| 10000 | 6.26139 | 6110 | 61.10 | 4516.30 |

**Supplementary Table S3 Sensitivity values from the case study**

| VAF | 100X | 200X | 400X | 600X | 800X |
| --- | --- | --- | --- | --- | --- |
| 0-0.01 | 0 | 0 | 0 | 0.051 | 0 |
| 0.01-0.02 | 0 | 0.009 | 0.140 | 0.281 | 0.386 |
| 0.02-0.03 | 0 | 0.034 | 0.483 | 0.720 | 0.856 |
| 0.03-0.04 | 0 | 0.292 | 0.934 | 1 | 1 |
| 0.04-0.05 | 0.038 | 0.667 | 0.990 | 1 | 1 |
| 0.05-0.06 | 0.304 | 0.922 | 1 | 1 | 1 |
| 0.06-0.07 | 0.568 | 1 | 1 | 1 | 1 |
| 0.07-0.08 | 0.935 | 1 | 1 | 1 | 1 |
| 0.08-0.09 | 0.960 | 1 | 1 | 1 | 1 |
| 0.09-0.10 | 0.990 | 1 | 1 | 1 | 1 |
| 0.10-0.11 | 1 | 1 | 1 | 1 | 1 |

**References**

1. Li H, Handsaker B, Wysoker A, Fennell T, Ruan J, Homer N, et al. The Sequence Alignment/Map format and SAMtools. Bioinformatics. 2009;25(16):2078-9.

2. Li H, Durbin R. Fast and accurate short read alignment with Burrows-Wheeler transform. Bioinformatics. 2009;25(14):1754-60.
